# Baseline bone health status in multi-ethnic South African postmenopausal breast cancer patients at initiation of aromatase inhibitor therapy: A descriptive study

**DOI:** 10.1371/journal.pone.0214153

**Published:** 2019-04-02

**Authors:** Karin J. Baatjes, Maritha J. Kotze, Micheal McCaul, Magda Conradie

**Affiliations:** 1 Department of Surgical Sciences, Faculty of Medicine and Health Sciences, Stellenbosch University, Tygerberg, South Africa; 2 Division of Chemical Pathology, Department of Pathology Faculty of Medicine and Health Sciences, Stellenbosch University and the National Health Laboratory Service, Tygerberg Hospital, Tygerberg, South Africa; 3 Biostatistics Unit, Division of Epidemiology and Biostatistics, Department of Global Health, Stellenbosch University, Tygerberg, South Africa; 4 Division of Endocrinology, Department of Medicine, Faculty of Medicine and Health Sciences Stellenbosch University, Tygerberg, South Africa; Fondazione Toscana Gabriele Monasterio, ITALY

## Abstract

**Introduction:**

Osteoporosis (OP) risk factor assessment and bone mineral density (BMD) testing are frequently omitted at baseline in aromatase inhibitor (AI) studies, which may lead to misinterpretation of AI associated bone loss. The present study describes bone health of South African postmenopausal women of predominantly Mixed Ancestry, prior to AI treatment.

**Methods:**

This descriptive baseline study, nested in a prospective AI cohort study, included postmenopausal women with endocrine sensitive breast cancer, aged 50 to 80 years. A baseline questionnaire documented demographic-, medical-, lifestyle- and fracture history. Body weight was assessed clinically, and body composition and BMD measured via dual energy absorptiometry (DXA). Data was analysed in STATA 14 using descriptive and inferential statistics.

**Results:**

101 participants were recruited, with a mean age of 61±7 years. Nearly a third (n = 32) of women at baseline fulfilled global criteria for bone protection (BMD T-score ≥-2SD (n = 18); BMD T-score -1.5SD to < -2SD with risk factors (n = 14). Lower body weight, body mass index (BMI), fat mass index and lean mass index were significantly associated with the participants with a BMD measurement in keeping with a diagnosis of OP (p <0.001). Low vitamin D was present in 93% of the cohort tested (n = 95), whilst deficient vitamin D status (<20ng/ml) was documented in 52 women (55%).

**Conclusions:**

In this study, a third of postmenopausal women considered for AI therapy fulfilled international criteria for bone protective pharmacological intervention. This emphasizes the need for clinical risk and BMD assessment in postmenopausal breast cancer patients at baseline. Body composition and bone health associations highlight bone fragility associated with lower body weight.

## Introduction

The increased risk for osteoporosis in postmenopausal women relates to physiological changes in the ageing female body. Osteoporosis (OP) can cause severe impairment of function and quality of life in elderly women due to the increased fracture risk, especially of the spine and hip [1,2]. Bone mineral density (BMD) measured by dual energy X-ray absorptiometry (DXA) is a two-dimensional measure of mineral content in specific skeletal regions [3]. It evaluates BMD changes over time and can assess response to therapeutic interventions [4,5]. The risk of fracture increases with a decline in BMD [1,6].

Endocrine treatment is indicated for estrogen receptor (ER) positive breast cancers. Tamoxifen, a selective estrogen receptor modulator has been used to treat endocrine responsive breast cancer for decades [7]. It protects against accelerated postmenopausal bone loss, by maintaining selective estrogenic effects on skeletal tissue [8]. Currently, aromatase inhibitors (AIs) are the gold standard in the treatment of endocrine sensitive breast cancer with an improved clinical outcome compared to Tamoxifen [9,10].

In postmenopausal breast cancer patients, aromatase enzyme inhibition decreases the already low levels of estrogen by blocking the conversion of androgen precursors to estrogen [11,12]. Numerous studies have documented accelerated bone loss and facture risk in postmenopausal women on AI therapy, affecting predominantly the axial skeleton [7,9]. The most pronounced loss is noted in the first two years of AI treatment and in early menopause (< 4 years).

Many conventional risk factors for the development of OP have been identified [13]. Age, gender, genetic predisposition and ethnic origin represent the most important non-modifiable risk factors. Modifiable factors such as low body weight, sedentary lifestyle, poor calcium nutrition and deficient vitamin D levels, smoking and alcohol excess, may also impact on bone density. Body weight is one of the most important determinants of BMD at most skeletal sites in women of all ethnicities [13,14].

In randomised controlled trials of AIs, the baseline state of BMD was not always reported. Similarly, conventional risk factors for fracture were not consistently quantified and the prevalence of osteoporotic fractures prior to AI therapy remains unknown [15]. The presence of conventional risk factors for osteoporosis and baseline BMD must be considered to accurately calculate the fracture risk attributable to AI therapy.

The study describes the baseline bone health status, prior to initiation of AI’s, in a multi-ethnic postmenopausal cohort with endocrine responsive breast cancer, resident in the Western Cape Province of South Africa. The associations between BMD and body composition as well as lifestyle factors, known to potentially adversely affect bone mineral status, were examined.

## Materials and methods

### Study population

This descriptive study, nested within a larger prospective cohort study was conducted at the tertiary breast clinic of Tygerberg Hospital, affiliated to the University of Stellenbosch. Postmenopausal women with newly diagnosed, endocrine sensitive breast cancer, stage 0-III were eligible for study entry. All 50–80 year old women were consecutively enrolled from August 2014 until February 2017. Race determination was made by self-declaration. Patients with known metabolic bone disease, and those with diseases (other than breast cancer), or medication known to adversely affect BMD were excluded from entry. The research was approved by the Ethical Review Board of the Faculty of Medicine, University of Stellenbosch (S13/05/103).

### Demographics

A demographic questionnaire administered at baseline, included age, family medical history, personal health, lifestyle, reproduction, falls and fractures. Lifestyle questions included the use of alcohol, smoking and activity level. The use of hormonal contraception and years since menopause (YSM) were documented. A history of prior fragility fractures, fall propensity and prolonged immobilization (>1 month) [13] was obtained.

### Anthropometry

Basic anthropometric measurements (weight, height, waist and hip circumference) were taken. Body mass index (BMI) values were divided into weight categories according to the World Health Organisation’s (WHO) classification [16].

### Densitometry

The DXA Hologic Discovery-W, S/N 70215; software Version 13.1 was employed to measure BMD and body composition. During the course of the study, coefficients of variation for BMD were <1.5%. The availability of a single and experienced DXA technician for the duration of the study is noteworthy. It ensured an excellent performance and a low intra-operative variability (<1%) of reported BMD measurements at all skeletal sites in patients initiated on AI-therapy.

### Body composition

Whole body DXA was used to measure and calculate total fat percentage, fat mass index (FMI), lean mass index (LMI), appendicular skeletal muscle mass as well as an android/gynoid fat ratio. Fat mass and lean mass measured by DXA are normalized for height to calculate a FMI (fat/height^2^), a measure of obesity and a lean mass/height^2^ as an index of total muscle mass. The appendicular skeletal muscle mass normalised for height^2^, is a good surrogate marker of sarcopenia, if found to be low. No local normative data for DXA measured body composition exist. The National Health and Nutrition Examination Survey (NHANES) reference dataset was thus used [16]. In the present NHANES database, fat comprises approximately 38% of body weight in females at age 25 years and is thus regarded as the normal reference value for our study. The FMI was used to categorize participants into weight classes similar to BMI (low/normal fat, excess fat, obese and morbidly obese]. The normal FMI range is 5–9 kg/m^2^, excess fat 9.1–13 kg/m^2^, obese 13.1–21 kg/m^2^ and morbid obesity indicated by a FMI in excess of 21 kg/m^2^. LMI and appendicular lean mass/height^2^ was categorized as being 2SD below or above expected with the cut-off values being 12.5 kg/m^2^ and 4.36 kg/m^2^, respectively.

### Bone mineral density

Femoral neck (FN), total hip (TH) and lumbar spine (LS) BMD were measured. No normative data for South African women of mixed or black race exist. We therefore, as currently recommended, used a uniform normative database for white women to calculate T-scores and to define osteopenia and osteoporosis for all ethnicities. A lateral vertebral assessment (LVA) detected prevalent morphometric vertebral fractures.

### Biochemistry

Morning blood samples were drawn to evaluate calcium homeostasis (serum calcium, phosphate, parathyroid hormone (PTH) and 25-OH Vitamin D levels) and to assess biochemical bone turnover (serum bone specific Alkaline Phosphatase (bALP) and Beta-CrossLaps/serum assay). Bone specific Alkaline Phosphatase (bALP) was measured with the Beckman Coulter Unicel DXI 800, a one-step immunoenzymatic assay. The normal reference range for this specific bALP assay in postmenopausal women is ≤ 22mcg/L. Beta-CrossLaps testing was performed using the Roche Cobas 6000 e601 analyzer and the Roche Beta-CrossLaps assay, a 2-site immunometric (sandwich) assay using electrochemiluminescence detection. This assay specifically detects crosslinked isomerized type I collagen fragments, independent of the nature of the crosslink. The upper range of normal for postmenopausal women is set at 1008 pg/ml. Bone turnover markers are frequently used in clinical trials and provide valuable information on the efficacy of osteoporotic treatments, but their predictive value is limited by their biological variation and they are not routinely used for individualized patient care and diagnosis.

### Statistical analyses

Data management and analysis were conducted in STATA 14. Descriptive statistics were used to summarise the data including baseline characteristics and outcomes. Continuous data were tested for normality using descriptive statistics (e.g. histograms) where normally distributed data were presented as means and standard deviations or as medians and interquartile ranges (IQR), for non-normally distributed data. Categorical data were presented as proportions and 95% confidence intervals. The associations between biological parameters and BMD were determined using one-way ANOVA and chi^2^ tests. To account for confounding, significant univariate predictors were included in a final multinomial logistic regression model at p<0.2 An alpha of 0.05 was considered statistically significant. Associations were reported as relative risks with 95% confidence intervals. Missing data were assumed to be missing at random and no inputting performed.

## Results

### Clinical demographic characteristics

A hundred-and-one postmenopausal participants were enrolled with a mean age of 61±7 years. Nearly half (n = 48) the women were 50–59 years of age, with only a minority of the cohort being 70 years and older (10%). Eighty-two percent of the study population were of Mixed Ancestry, reflecting the hospital’s reference population. Caucasians represented 13.4% of the total cohort and only two Black and three Indian women were included. Demographics and lifestyle data are presented in Table 1.

**Table 1:** Summary of lifestyle and menstrual data of breast cancer patients at baseline.

Clinical characteristics (n = 101)	
**Age (years)**	**61 ± 7**
• 50–59 yrs	48 (48%)
• 60–69 yrs	43 (43%)
• 70 yrs^+^	10 (10%)
**Smoking**	
• ever	42 (42%)
• current	28 (28%)
**Alcohol**	
• abstain	79 (79%)
• 1–7 units per week	22 (22%)
• >7 units per week	0
**Activity level**	
• Indoors	20 (20%)
• Indoors and Outdoors	81 (80%)
**Falls in last year**	
• Any fall	0
**Clinical fractures**	
• non-vertebral	7 (7%)
**Family history of OP**	
• positive	1 (1%)
**Age at menopause (years)**	**48 ± 5 years**
**Duration of menopause (n = 72)**	**12 ± 8 years**
• 0–4 yrs	15 (21%)
• 5–10 yrs	15 (21%)
• > 10 yrs	42 (58%)
**Hot Flashes**	
• ever	64 (63%)
**Hormonal contraception (ever)**	
• Depo-Provera	19 (19%)
• OCP	30 (30%)

Values for age, age at menopause and duration of menopause expressed as mean ± SD, rest of data expressed as n (%). Cohort n = 101 for all clinical characteristics tabulated unless otherwise specified. OP = osteoporosis, OCP = oral estrogen containing contraceptive preparation

Nearly half of the study population (42%) smoked at some stage in their lives and 28% of women were current smokers. Alcohol consumption was minimal with abstinence reported by 79%. No intake in excess of 7 units per week was reported. Most women lead a moderately active lifestyle with indoor and outdoor activities documented in 80%. No falls were reported, seven women sustained a previous non-vertebral fracture and one participant reported a family history of osteoporosis. Menopause occurred at a mean age of 48±5 years with a short duration (≤5years) in 21%. Hormonal contraception was used by 49 women, 19 of whom used an injectable progesterone-only preparation only.

### Mineral homeostasis, calciotropic hormones and biochemical bone turnover markers

Normal vitamin D status was only documented in 7% of the cohort. Insufficient vitamin D levels (20-30ng/ml) were present in 38% (n = 36), whilst deficient vitamin D status (<20ng/ml) was documented in 55% (n = 52). Bone disease caused by vitamin D deficiency is usually associated with values below 10-12ng/ml. This severe deficiency was only evident in two participants, of whom both had a normal BMD. Despite the almost universal 25-OH-Vitamin D deficiency, only 24 subjects (25%) had secondary hyperparathyroidism. Bone specific alkaline phosphatase (n = 98; range 4.4–26.5mcg/L; marginally abnormal values in 4 participants 23.6, 24.3, 24.3 and 26.5mcg/mL) and β-cross laps (n = 70; range 90–1340 pg/mL; one abnormal value of 1340 pg/mL) were normal in 96% and 99% of the cohort respectively. Biochemical bone turnover assessment ruled out any significant baseline increase in bone turnover in almost all study participants.

### Body composition

Clinical and densitometric parameters of body composition are tabulated in Table 2. A concerning 85% of women were overweight, with 59% in the obese categories of WHO-BMI [16]. A waist/hip ratio in excess of 0.85 was present in the majority (78%). Densitometric assessment of body composition was in accordance with our clinical assessment. The mean total body fat mass percentage (45 ± 6%) and the mean fat mass index (FMI) (14.6 ± 4.9kg/m^2^) was significantly above normal. A high FMI was documented in 89% and a FMI indicative of obesity was present in 59% of the cohort.

**Table 2:** Body composition in postmenopausal breast cancer patients at baseline.

**Clinical parameters (n = 101)**	**Category values**	**Measurement**
Weight (kg)		81.2 ± 19.4
Height (cm)		158.6 ± 6.0
BMI (kg/cm^2^)		32.4 ± 7.8
BMI weight categories		
• Low/normal body weight	≤ 25 kg/m^2^	15 (15%)
• Overweight	25.1–29.9 kg/m^2^	26 (26%)
• Obesity	30–39.9 kg/m^2^	42 (41%)
• Morbid obesity	≥40 kg/m^2^	18 (18%)
Waist circumference (cm)		102.1 ± 15.8
Waist/Hip circumference (cm)		0.9 ± 0.1
• > 0.85*		79 (78%)
**Densitometric parameters (n = 101)**	**Category values**	**Measurement**
**Mean Total Body Fat Mass (%)**		**45 ± 6**
• Normal	≤ 38%	6 (6%)
• Increased	> 38%	95 (94%)
**Mean Fat Mass Index (FMI) (kg/m**^**2**^**)**		**14.6 ± 4.9**
• Normal	5–9	11 (11%)
• Excess fat	9.1–13	30 (30%)
• Obese	13.1–21	53 (52%)
• Morbid obesity	>21	7 (7%)
**Mean Lean Mass Index (LMI) (kg/m**^**2**^**)**		**16.7± 2.8**
• Above third centile (>2SD)	≥ 12.5	98 (97%)
• Normal range	< 12.5	3 (3%)
**Mean Appendicular Lean mass/height**^**2**^ **(kg/cm**^**2**^**)**		**7.2 ± 5.5**
• Above third centile (>2SD)	≥ 4.36	99 (98%)
• Normal range	< 4.36	2 (2%)
**Mean Android/Gynoid ratio**		**1.0 ± 0.1**
• Gynoid dominant fat distribution	≤ 1	44 (44%)
• Android dominant fat distribution (visceral)	> 1	57 (56%)

*Body composition evaluated clinically and with DXA*. *Mean values presented as means* ± *standard deviation unless otherwise specified*. *Cohort sub-classified into WHO weight categories based on BMI and percentage of cohort with waist/hip circumference indicative of metabolic syndrome* [16,17] noted*. *Cohort also sub-classified into Fat Mass Index classification ranges in accordance with BMI weight categories*. *LMI and appendicular lean mass/height*^*2*^ *divided into categories below and above the third centile (2SD) for specific measurement in young NHANES females*. *All densitometric measured categories defined based on NHANES data base for young normal females within normal BMI range* [16] *Data expressed as n(%) of subjects within all weight categories*.*Bone mineral density*

Lean mass appeared well maintained. A significantly decreased lean mass index (LMI) (< 12.5 kg/m^2^) and appendicular lean mass/ height^2^ (<4.36kg/cm^2^) indicative of a loss of muscle strength, was documented in only three and two subjects respectively.

The clinically determined BMI and the densitometric FMI were remarkably similar in the classification of women within the different weight categories. In the normal to low BMI category, three women had a FMI marginally above 9 (10.3 kg/m^2^ in two subjects and 10.5 kg/m^2^ in the third subject), and only four women in the obese category, had a FMI below 13.1 kg/m^2^ (6.7%).

Baseline BMD was assessed at the lumbar spine in all participants (n = 101) and in 100 women at the femoral neck and total hip region (bilateral hip replacement in one participant) (Table 3). BMD is expressed as an absolute density in g/cm^2^ and the deviation from expected peak value for the specific individual i.e. as a T-score to determine the patient’s specific BMD category as either within the normal, osteopenic or osteoporotic range[18]. The mean BMD at all the measured sites for the total cohort was within the normal range (T-scores less -1 SD below expected peak).

**Table 3:** Bone Mineral Density in postmenopausal breast cancer patients at baseline.

BMD	Lumbar Spine BMD (n = 101))	Femoral Neck BMD (n = 100)	Total Hip BMD (n = 100)
**Absolute value g/cm^2^**	0.982 ± 0.171	0.780 ± 0.119	0.913 ± 0.142
**T-score**	-0.5 ± 1.6	-0.65 ± 1.1	-0.2 ± 1.1
• **Normal**	58 (58%)	61 (61%)	75 (75%)
• **Osteopenia (< −1.0 > -2.5)**	30 (30%)	32 (32%)	21 (21%)
• **Osteoporosis (≤ -2.5)**	**13 (13%)**	**7 (7%)**	**3 (3%)**
• **High BMD (> 2.5)**	3 (3%)	1 (1%)	1 (1%)

*Absolute BMD values and T-scores at all the measured sites are presented as means* ± *standard deviation for the total study population*. *The cohort is then sub-classified into WHO-BMD categories* [19]. *Number of patients and percentage of study population within subgroups for all measured sites is noted*. *Normal subgroup also include patients with measured BMD T-score more than + 2*.*5SD*

A normal BMD at all measured sites was documented in 48 (48%) of the total study population. A concerning 52% of participants thus displayed osteopenia or osteoporosis at one or more measured sites i.e. BMD T-score deviations of -1 SD or more. BMD in keeping with osteoporosis at one or more sites was present in 14 women (14%) prior to any hormonal intervention for breast cancer. Osteopenia and osteoporotic range BMD were most prevalent in the axial skeleton (Table 3).

DXA-LVA indicated mild morphometric vertebral abnormalities in 15 women. Conventional lumbosacral X-rays excluded significant vertebral compression (≥20% of vertebral height) in all these women.

### Clinical characteristics, body composition and biochemistry within BMD subcategories (normal, osteopenia, osteoporosis)

Chronological age was similar in the BMD subcategories. Years since menopause (YSM) increased with worsening bone profile, with a mean duration of menopause 5 years longer in those with OP compared to the normal BMD subgroup. No significant association between YSM and BMD subgroups was noted (p = 0.14). In all the patients with OP, in whom the duration of menopause was documented (n = 6), YSM exceeded 5 years.

Body composition differed amongst BMD subcategories, with significantly lower total body weight, BMI, FMI, total fat percentage and LMI documented in the women with OP (p<0.001; Table 4). Fifty percent of women (7/14) with baseline OP had a low/normal BMI of ≤25 kg/m^2^. The rest were overweight (n = 4) or obese (n = 3). No morbidly obese woman was osteoporotic.

**Table 4:** Body composition within DXA-BMD subcategories.

Clinical characteristics, body composition and biochemistry	BMD subcategories			p-value
	Normal (n = 50)	Osteopenia (n = 37)	Osteoporosis (n = 14)	
*Clinical characteristics*				
Age (yrs)	59 ± 6	62 ± 7	64 ± 8	0.074
Years since menopause (yrs)	11 ± 9	14 ± 3	16 ± 6	0.14
*Body composition*				
Total body weight (kg)	86.6 ± 17.3	79.5 ± 19.2	63.8 ± 15.4	<0.001
BMI (kg/m^2^)	34.3 ± 6.4	31.7 ± 8.4	25.8 ± 6.2	<0.001
Waist/Hip ratio	0.91 ± 0.06	0.88 ± 0.08	0.91 ± 0.14	0.48
Total body fat (%)	46.9 ± 5.0	44.9 ± 6.6	41.6 ± 5.9	0.144
FMI (kg/m^2^)	15.0 ± 4.3	14.0 ± 4.9	10.5 ± 3.6	<0.001
LMI (kg/m^2^)	17.6 ± 2.5	16.3 ± 2.9	14.2 ± 1.9	<0.001
Appendicular lean mass (kg/m^2^)	8.1 ± 7.6	6.6 ± 1.1	5.5 ± 1.1	0.19
*Biochemistry*				
Vitamin D (ng/ml)	19.8 ± 5.8	18.9 ± 6.3	22.1 ± 8.4	0.290
• Total n	49	35	11	
• Sufficient [>30ng/ml] n (%)	3 (6%)	2 (5%)	2 (18%)	
• Insufficient [20-30ng/ml] n (%)	21 (43%)	12 (34%)	3 (27%)	
• Deficient: [< 20ng/ml] n (%)	25 (51%)	21 (60%)	6 (54%)	
PTH (pmol/L)	5.54 ± 2.87	5.10 ± 1.99	6.77 ± 4.36	0.221
• Total n	48	36	11	
• normal [1.6–6.9 pmol/L] n (%)	36 (75%)	29 (80%)	6 (54%)	
• elevated n (%)	12 (25%)	7 (20%)	5 (46%)	

All values expressed as means ± SD, unless otherwise specified. Yrs = years; BMI = body mass index; FMI = fat mass index; LMI = lean mass index; BMD = bone mineral density. BMD subcategories refers to DXA BMD T-score: normal = less than 1SD below norm, osteopenia = -1 to -2.49 SD below normal and osteoporosis = ≥-2.5 SD below norm. p- value significant at < 0.05 for continuous comparison normal BMD versus osteopenia and osteoporosis subgroups.

The mean 25-OH-Vitamin D (p = 0.290) and PTH levels (p = 0.221) were similar in the BMD subcategories (Table 4). Nearly half (46%) of women with OP had compensatory secondary hyperparathyroidism, but a significant adverse BMD effect in ascending PTH tertials, could not be demonstrated (p = 0.720).

**Fig 1:**
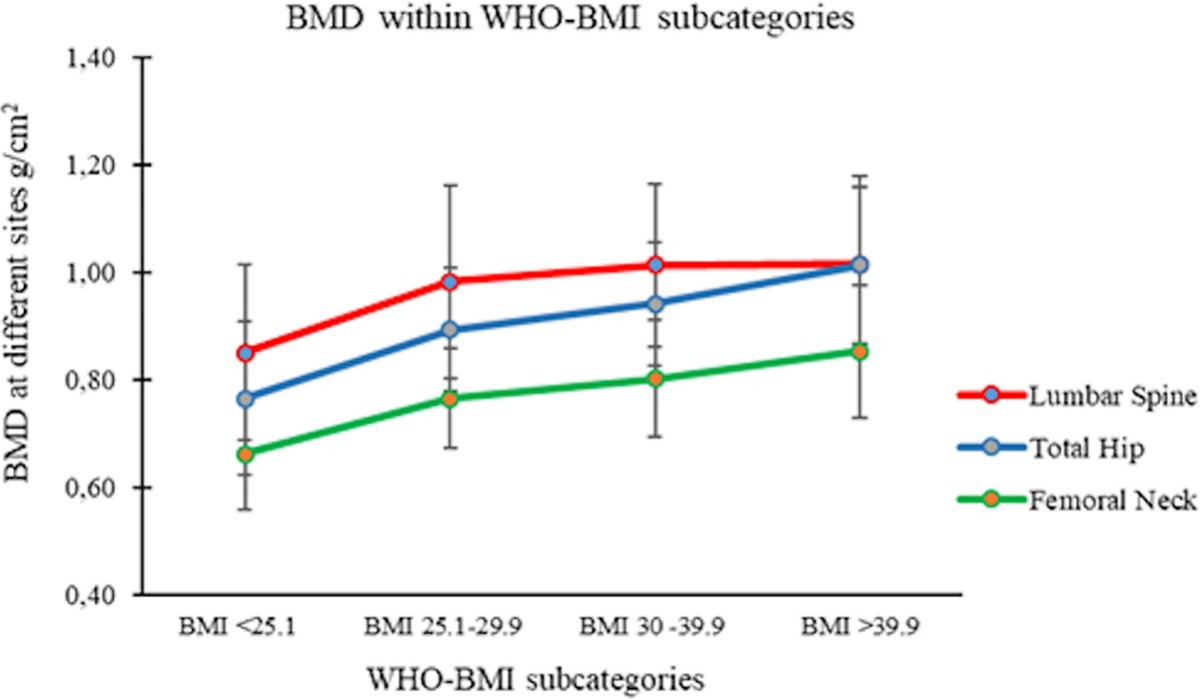
Bone mineral density within the WHO-BMI subcategories.

When looking at BMD subcategories at specific bone sites i.e. at the femur neck, total hip and lumbar spine, a similar trend was noted for clinical characteristics, body composition and biochemistry relationships compared to the composite BMD. BMD at all measured sites increased significantly with increasing BMI based on WHO subcategories as demonstrated in Fig 1.

Multinomial regression adjusting for known confounders indicated PTH (RR 1.61, 95% CI 1.15–1.25) and LMI (RR 0.3, 95% CI 0.11–0.85) were significantly associated for OP compared to normal bone status (Table 5).

**Table 5:** Crude versus adjusted predictors (reporting relative risk) for BMD status at baseline.

	Crude RR		Adjusted RR	
Predictors of BMD	Osteopenia	Osteoporosis	Osteopenia	Osteoporosis
**BMI**	0.95 (0.83–1.01)	0.82 (0.77–0.92)	1.14 (0.90–1.44)	1.24 (0.80–1.93)
**Fat mass index**	0.91 (0.83–1.00)	0.74 (0.62–0.88)	0.87 (0.66–1.14)	0.73 (0.44–1.21)
**Lean mass index**	0.82 (0.69–0.98)	0.54 (0.35–0.75)	0.70 (0.47–1.03)	0.30* (0.11–0.85)
**PTH**	0.93 (0.79–1.10)	1.13 (0.92–1.38)	0.98 (0.81–1.17)	1.61* (1.15–2.25)

Comparison of predictors of normal BMD. RR (95% CI)

*p-value significant (adjusted only) at <0.05.

## Discussion

In this study a concerning 14% of women had osteoporosis and half of the cohort were osteopenic prior to any intervention. Body composition, especially lean mass, was positively associated with bone mass at all measured sites and the risk of being osteoporotic less with increasing Lean Mass Index (p<0.0001). Clinical and densitometric measures of body weight and composition were universally lowest in women with OP (p<0.001).

Only seven participants had sufficient 25-OH Vitamin D levels with compensatory, secondary hyperparathyroidism documented in more women with OP (45%) than in the rest of the cohort (23%).

The addition of AIs in the endocrine treatment of breast cancer compounds OP risk. The International Osteoporosis Foundation Bone and Cancer Working Group indicates that women on AI therapy for breast cancer, experience a two to four-fold increase in bone loss [20]. Clinical trials show an approximately 10% increase in absolute fracture risk for women on AI therapy [12,18]. The fracture incidence in women with breast cancer on AI therapy was reported to be 18–20% after five years of follow-up [21]. It is thus essential to delineate BMD and clinical risk factors for bone loss and fracture at the start of AI treatment to allow for risk stratification and appropriate preventative measures. Breast cancer treatment seeks not only to prolong survival, but also to limit side effects [12,22].

BMD is viewed as the most robust indicator of fracture risk in untreated patients [3]. Ethnic differences in bone mass and fracture risk have been described globally, but established data mostly limited to black and white populations [2,23]. More than 80% of our study population were of Mixed Ancestry, a population subgroup in whom BMD and fracture prevalence is largely unknown. In the only reported data from South Africa, BMD measurements in women of Mixed Ancestry were similar to whites at all measured sites [24]. Extrapolated from densitometric studies, fracture risk is thus expected to be similar in white women and those of Mixed Ancestry. No formal study looking at fracture prevalence in women of Mixed Ancestry in SA has, however, been conducted to date and the prevalence of vertebral fractures in this ethnic group thus remains unknown. Such knowledge will facilitate management strategies for osteoporosis and fractures across all ethnic groups and in the post-menopausal woman treated for breast cancer [25].

The lowered BMD in more than half of our cohort at baseline is concerning. This argues for routine BMD measurements in postmenopausal women of Mixed Ancestry with breast cancer considered for AI therapy. In the majority of our subjects, significant bone loss was confined to the axial skeleton, inferring an increased vertebral fracture risk. Global consensus recommends that all women with a BMD T-score ≤ -2 SD at any measured site, should undergo bone protective therapy [20]. Additionally, patients with a BMD T-score between -1.5SD and -2SD with one or more additional risk factors for bone loss, should also be considered for treatment. These include age above 65 years, smoking, a family history of hip fracture or a personal history of fragility as well as low body weight and a course of steroid therapy in excess of 3 months.

Based on these recommendations, eighteen women in our cohort (18%) warranted bone specific therapy based on BMD per se. An additional fourteen participants also required protection as they had a BMD T-score between -1.5 SD and -2 SD and at least one conventional risk factor for bone loss. Nearly one-third (32%) of women at baseline thus fulfilled the criteria for bone specific intervention.

Obesity is associated with higher BMD in postmenopausal women and slower rates of bone loss at the hip and spine. Eighty-five percent of our cohort was overweight, a factor that may afford bone protection. In continuous comparison, the relationship between fat mass indices and lean mass indices with BMD measurements, were similar in this study. Fat mass has shown positive association with BMD due to increased mechanical loading and the release of osteogenic hormones from adipose tissue [26]. The production of androgens is higher in obese than in normal weight women [27]. However, fat mass also produce inflammatory cytokines, which may negatively influence BMD [28]. Skeletal muscle mass has consistently been associated with increased BMD in all women due to the mechanical forces on bone during muscle activity [4,29]. A positive relationship between all body composition parameters and BMD at spine and hip regions were documented in our study and in another SA study in healthy black and white women [30]. A beneficial effect of both increased fat and lean mass on BMD maintenance was thus noted irrespective of site and ethnicity.

A low/normal BMI was present in fifty percent of osteoporotic women in sharp contrast with the total cohort in whom 85% were obese. Low body weight is a well-established risk factor for OP in breast cancer patients and also reflects in our study [16,31]]. However, overweight and obese BMI did not preclude OP in this cohort.

Low vitamin D is a known risk factor for osteoporosis due to the associated negative calcium balance and compensatory secondary hyperparathyroidism with increased bone resorption [32–34]. The vast majority of our study participants (93%) had insufficient or deficient vitamin D levels, an extremely high and concerning figure. A marked seasonal variation in vitamin D3 production was previously documented in Cape Town, with very little being formed during the winter months [35]. Increased skin pigment and obesity are well known risk factors for decreased cutaneous vitamin D production, both present in the majority of our study subjects. Mean Vitamin D levels did not, against expectation, differ amongst the BMD subcategories (p = 0.290). This may be explained by the almost universal Vitamin D insufficiency in study participants.

The American Society of Clinical Oncology considers AI-therapy as the gold standard treatment for hormone-receptor-positive breast cancer. Baseline assessment of bone health (incorporating a BMD assessment), timeously identifies women at risk of fracture and ensures appropriate preventative strategies. Fragility fractures, with its associated morbidity and even mortality, may significantly impact on the quality of life of AI-treated individuals.

Conclusions drawn from this study are limited by the small sample size of 101 women. Data obtained nonetheless contributes to the limited knowledge pool regarding the baseline bone health of South African postmenopausal women of diverse ethnicity with breast cancer. Another limitation of this work is the absence of a healthy control group. Data derived from this study are thus not able to delineate differences in bone health between breast cancer participants and healthy postmenopausal women. Our data, however, does argue for optimal assessment of bone health, including a BMD, in postmenopausal women of Mixed Ancestry considered for therapy with potential adverse bone effects. Conversely, the strength of our study is that densitometric data were obtained by making use of a single, very experienced densitometrist that positively impacts on the validity of both our body composition and BMD data.

## Conclusion

A significant 32% of postmenopausal women considered for AI therapy fulfilled criteria for bone protection. Data obtained in this study emphasizes the absolute need for bone risk assessment in all postmenopausal women with breast cancer who are considered for AI therapy. Valuable information regarding the relationship between body composition variables and bone health of postmenopausal women of Mixed Ancestry residing in the Western Cape Province of South Africa was generated. Improved insights into ethnic variations of bone health, provided by studies such as ours, will enable preventive approaches to osteoporosis for post-menopausal women on breast cancer treatment. Ultimately, it may also inform local health policy.

## Supporting information

S1 Dataset (XLSX)
